# Development and Validation of the Brief Inventory of Treatment Expectations in Chronic Pain (BITEC)

**DOI:** 10.1002/ejp.70211

**Published:** 2026-01-29

**Authors:** Wolnei Caumo, Bárbara Regina França, Jaira Ehlers, Stela Maris de Jezus Castro, Rogério Boff Borges, Vania Naomi Hirakata, Graziele Borges Bueno, Iraci LS da Torres, Felipe Fregni

**Affiliations:** ^1^ Post‐Graduate Program in Medical Sciences, School of Medicine Universidade Federal do Rio Grande do Sul (UFRGS) Porto Alegre Brazil; ^2^ Laboratory of Pain and Neuromodulation at Hospital de Clínicas de Porto Alegre (HCPA) Porto Alegre Brazil; ^3^ Pain and Palliative Care Service at HCPA Porto Alegre Brazil; ^4^ Department of Surgery, School of Medicine UFRGS Porto Alegre Brazil; ^5^ Faculdade de Medicina, Programa de Pós‐Graduação Em Epidemiologia Universidade Federal do Rio Grande do Sul Porto Alegre Brazil; ^6^ Hospital das Clínicas de Porto Alegre Unidade de Bioestatística, Diretoria de Pesquisa Porto Alegre Brazil; ^7^ Department of Statistics, Institute of Mathematics and Statistics Universidade Federal do Rio Grande do Sul Porto Alegre Brazil; ^8^ Laboratory of Pharmacology of Pain and Neuromodulation, Experimental Research Center HCPA Porto Alegre RS Brazil; ^9^ Center for Clinical Research Learning, Physics and Rehabilitation Department Spaulding Rehabilitation Hospital Boston Massachusetts USA

**Keywords:** assessment, outcome expectancy, pain, placebo effect, scale development, validity tests

## Abstract

**Background:**

Expectations shape therapeutic outcomes, yet their systematic assessment remains limited in clinical and research settings. To address this gap, we developed and validated the Brief Instrument for the Assessment of Treatment Expectations in Chronic Pain (BITEC) using Item Response Theory.

**Methods:**

The study comprised four phases. (I) Twenty‐one items were generated from expectation constructs and refined to 11 through a Delphi review (≥ 80% agreement). (II) Comprehensibility was tested in 30 women with fibromyalgia, and the scale was applied to 484 chronic pain patients; items (0–10) were recoded into four categories, and IRT reduced them to nine. (III) The final version was administered to 1127 adults with chronic pain (79.3% fibromyalgia; 20.7% nociceptive/neuropathic), and latent‐class modelling defined low–high expectation cutoffs. (IV) Construct validity was assessed via discriminant analyses in calibration (*n* = 1127) and validation (*n* = 242) samples to evaluate whether BITEC levels differentiated diagnostic groups, pain impact and catastrophizing. (V) We developed a bedside app to support expectation‐level classification.

**Results:**

The nine‐item BITEC showed discrimination between high and low expectations (AUC 0.915; 95% CI 0.897–0.933; sensitivity 79%, specificity 96%). Across both samples, BITEC demonstrated construct validity, distinguishing expectation categories based on symptom severity, catastrophizing and pain burden. Expectation levels varied across pain phenotypes, decreasing from nociceptive pain (56.1%) to fibromyalgia (42.7%) and multiple pain conditions (26.9%). Higher symptom severity was associated with higher expectations.

**Conclusion:**

BITEC is a brief, reliable, theory‐grounded instrument for stratifying treatment expectations in chronic pain; applicability across treatment modalities and clinical contexts warrants further investigation.

**Significance Statement:**

Expectations strongly shape therapeutic outcomes but remain difficult to measure. The BITEC, a brief IRT‐based tool, offers a reliable way to classify treatment expectations in chronic pain, supporting personalised care and improving clinical decision‐making.

## 1 Introduction

Chronic non‐cancer pain affects about 19% of adults in Europe (Breivik et al. 2006) and is defined as non‐malignant pain persisting ≥ 3 months or beyond expected healing (Treede et al. 2015). Despite its prevalence, up to 79% of patients report inadequate control and 43% receive no treatment (Breivik et al. 2006). As a multidimensional and individualised experience, pain requires understanding patient expectations to support person‐centred care (Fitzcharles et al. 2021). Expectations arise from personal and social experiences and reflect biopsychosocial, cognitive and behavioural mechanisms (Colloca and Miller 2011). They predict treatment outcomes (Mondloch et al. 2001) and influence placebo and nocebo responses (Benedetti 2020; Schedlowski et al. 2015). Positive expectations modulate brain activity and symptom perception, whereas negative ones foster distress and maladaptive behaviours (Leventhal et al. 2016; Petrie and Weinman 2012). Clinicians shape these mechanisms through communication, goal setting and shared decision‐making (Kaptchuk and Miller 2015). However, expectations remain difficult to assess due to their dynamic, context‐dependent, multidimensional nature (Laferton et al. 2017). They encompass structural, process and outcome elements and include value and predictive dimensions (Kravitz 1996; Laferton et al. 2017; Thompson and Sunol 1995), both relevant to chronic pain.

Systematic reviews and meta‐analyses reveal marked heterogeneity in how expectations are defined and measured, many studies using single items or non‐validated measures, undermining reliability and construct validity (Auer et al. 2016; Bowling et al. 2012; Laferton et al. 2022, 2017; Haanstra et al. 2012; Zywiel et al. 2013). Although condition‐specific instruments—such as the Treatment Expectations in Chronic Pain Scale (Page et al. 2019)—capture important nuances, they hinder comparability across interventions (van Hartingsveld et al. 2010; Zywiel et al. 2013; Laferton et al. 2017). Broader tools developed in medical or psychological contexts (Younger et al. 2012; Laferton et al. 2017; Barth et al. 2019) also lack sensitivity to chronic pain features—including symptom persistence, disability, psychological comorbidities, catastrophizing and central sensitization. Even pain‐focused tools (Jose et al. 2017) omit cognitive, affective and behavioural components essential for understanding adherence and perceived control. These limitations highlight the need for instruments capable of assessing expectations within a personalised biopsychosocial framework and aligned with patient‐reported outcomes (PROs). Such tools must be theoretically grounded, clinically feasible and sensitive to latent traits underlying expectations in complex conditions like chronic pain. Item Response Theory (IRT) offers a robust approach for developing concise, valid and discriminative measures that enhance precision and clinical applicability.

This study aimed to develop and validate a psychometric instrument to assess treatment expectations in chronic pain patients. The process comprised four phases: *Phase I*—Item Development: generation and content validation of items using PROMs. *Phase II*—Scale Construction: refinement of items through clarity testing and IRT, and classification of expectation levels via latent class modelling. *Phase III*—Scale Evaluation: assessment of construct validity by determining whether BITEC distinguishes expectation levels across diagnostic profiles, pain severity, multidimensional pain impact and catastrophizing. *Phase IV*—App Development: creation of a digital tool to measure and classify expectations as low or high.

## 2 Participants and Methods

The study protocol received approval from the institution's ethics committee (application n^o^. Research Group and Postgraduate Studies (GPPG): 2021‐0062; Certificate of Ethical Appreciation Presentation (CAAE): 43834721.9.0000.5327 Postgraduate Research Group at Hospital de Clinicas de Porto Alegre, HCPA). Before participating in the study, all subjects provided written informed consent. The study was conducted from March 2022 to December 2024.

**FIGURE 1:**
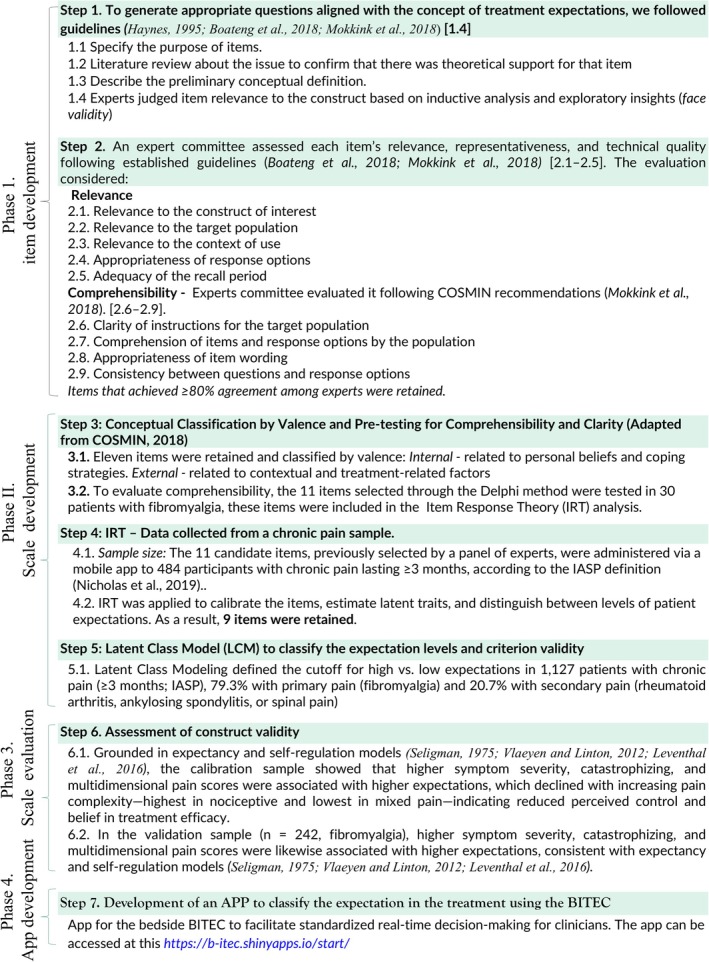
Phases of study development.

This study was conducted in three phases. *Phase I* involved the construction of items, focusing on developing the set of items for the eventual scale. *Phase II* focused on scale construction, *Phase III* involved scale evaluation and *Phase IV*—App Development. The sequence of the protocol is illustrated in Figure 1.

### 2.1 Phase 1: Item Development of Items of Brief Inventory of Treatment Expectations in Chronic Pain (BITEC)

#### 2.1.1 Phase I—Item Generation

The development of the BITEC followed a structured, theory‐driven process to ensure content validity, conceptual coherence and alignment with international PROM‐development standards. Grounded in an integrative model of treatment expectations (Laferton et al. 2017), the conceptual framework incorporated both cognitive and behavioural components of expectation—including beliefs about treatment efficacy, perceived control, engagement and anticipated behavioural responses. Items were designed for patients undergoing medical treatment for chronic pain (Pogatzki‐Zahn et al. 2019) and covered outcome‐, structural‐ and process‐related expectation domains.

##### 2.1.1.1 Step 1—Item Generation

As a Patient‐Reported Outcome Measure (PROM), the BITEC was conceived to capture treatment expectations across internal valence (e.g., personal beliefs, coping strategies, confidence, perceived control) and external valence (e.g., perceptions of treatment modality, contextual cues, care delivery). An initial set of 21 candidate items was generated to represent key theoretical domains, including anticipated benefits, perceived control, treatment intensity, side‐effect tolerance and procedural characteristics. This process followed established methodological recommendations (Terwee et al. 2018) and involved: (1) Defining item intent; (2) Conducting a focused literature review; and (3) Performing conceptual mapping and exploratory refinement to ensure theoretical and semantic adequacy.

##### 2.1.1.2 Step 2—Expert Content Validation (Delphi Method)

Face and content validity were established through 2–3 iterative Delphi rounds conducted by a multidisciplinary panel composed of psychologists, physicians certified in pain medicine, neuroscientists and rehabilitation professionals. Following COSMIN and Boateng et al. guidelines (2018), experts assessed each item for relevance, clarity, comprehensibility, representativeness of theoretical domains, and contextual fit for chronic‐pain populations. Items were rated on a four‐point equivalence scale, and those achieving ≥ 80% agreement were retained. After refinements incorporating expert feedback, 11 items (see Appendix S1) demonstrated satisfactory content validity and conceptual consistency. These items were advanced to Phase II for conceptual classification, cognitive pre‐testing and psychometric evaluation.

#### 2.1.2 Phase II—Scale Development

Phase II consisted of three sequential steps aimed at constructing the BITEC scale, including conceptual classification, cognitive evaluation, psychometric refinement, and derivation of the expectation‐level classification system.

##### 2.1.2.1 Step 3—Conceptual Classification by Valence and Pre‐Testing for Comprehensibility

The 11 items retained from Phase I were conceptually classified according to expectation valence, following the theoretical distinction between:

*Internal valence*—expectations grounded in personal beliefs, coping abilities, confidence and perceived control.

*External valence*—expectations related to contextual or treatment‐related elements, such as therapeutic modality, treatment intensity and the care process.

The preliminary version of the BITEC, composed of 11 items, was pre‐tested with 30 female patients diagnosed with fibromyalgia according to American College of Rheumatology (ACR) 2016 criteria (Wolfe et al. 2016). The goal was to evaluate the comprehensibility and clarity of each item using a verbal numerical scale from 0 (unclear) to 10 (completely clear). All items received average clarity scores ≥ 9. The sample had a mean age of 49.1 (SD = 12.0) and a mean education level of 11.9 years (SD = 3.3). Following this, the expert committee re‐evaluated the items using the Delphi method. Based on patient understanding and expert consensus, all 11 items were maintained with a standardised response scale from 0 (absence of content) to 10 (maximum intensity of content).

##### 2.1.2.2 Step 4—Item Response Theory (IRT) Calibration

The 11 items were administered through a mobile‐app platform to 484 literate adults aged 18–70 years with non‐oncological chronic pain lasting ≥ 3 months, meeting IASP criteria (Nicholas et al. 2019). Recruitment took place both in the Pain Service of the Hospital de Clínicas de Porto Alegre and in the community via public announcements, mass media and online platforms. Interested individuals accessed a secure REDCap link, provided electronic informed consent, and completed sociodemographic, clinical and symptom‐screening questionnaires. Up to three reminder messages were sent to those who initiated but did not complete registration, with a final reminder after 10 days.

The committee categorised items into four expectation domains: treatment expectations (benefits and side effects, items 1–3, 8 and 9); behavioural expectations (self‐efficacy and outcome, items 4, 5); generalised expectations (item 6); structural/process expectations (7). Additional technical details are provided in Appendix S1.

Item calibration was performed using the Samejima Graded Response Model (Samejima 1969, 1997), which enabled the estimation of latent traits and assessment of item discrimination. Based on their psychometric properties, nine items were retained for their ability to capture the most relevant information about the underlying construct of treatment expectations. Based on psychometric performance and discrimination capacity, nine items were retained. Items 10 (‘past experiences’) and 11 (‘dynamic expectations’) were excluded because they did not meaningfully improve latent‐trait discrimination. The items were recoded into four ordered categories: never (0), sometimes (1–3), almost always (4–7) and always (8–10).

The retained items map onto core expectation domains grounded in a personalised biopsychosocial framework: *Biological domain*: beliefs about analgesic efficacy, treatment strength, control and adequacy (Items 1, 3, 5 and 9); *psychological domain*: hope, tolerance, commitment and metacognitive expectation processes (Items 2, 6, 7 and 8); *social/interactional domain*: doctor–patient interaction (Item 4).

These nine items, modelled through the Graded Response Model, represent a continuous latent variable with a mean of zero and standard deviation of one. Interpretation is based on the number of standard deviations above or below the mean, reflecting the individual's level of treatment expectation relative to the sample. The final items are presented in Table 1. Model fitting was conducted using the ltm package (version 1.2–0) in R.

**TABLE 1:** BITEC: A Set of items selected by the IRT (*n* = 484).

The response options for the items were classified into four categories				
1. I believe that this treatment will help reduce my pain	Never	Sometimes	Almost	Always
2. I hope that this treatment contributes to improving my quality of life	Never	Sometimes	Almost	Always
3. I believe that controlling my pain depends on this treatment	Never	Sometimes	Almost	Always
4. My confidence in the treatment depends on how I am treated by the doctor	Never	Sometimes	Almost	Always
5. I believe stronger treatment leads to better pain relief	Never	Sometimes	Almost	Always
6. I believe I can continue the treatment even if I experience side effects	Never	Sometimes	Almost	Always
7. I am committed to my treatment regardless of the difficulties I face	Never	Sometimes	Almost	Always
8. I perceive that my expectations influence the effects of the treatment	Never	Sometimes	Almost	Always
9. I believe this treatment has the ideal strength to relieve my symptoms	Never	Sometimes	Almost	Always

##### 2.1.2.3 Step 5: Latent Class Model (LCM) to Classify the Expectation Levels and Criterion Validity

Individuals aged 18 years or older with a diagnosis of chronic pain, defined according to the International Association for the Study of Pain (IASP) criteria, as pain persisting or recurring for more than 3 months. This operational definition ensured that all participants met the internationally accepted standard for chronic pain duration, consistent with the ICD‐11 classification (Nicholas et al. 2019). Participants were recruited through public announcements and online platforms such as Facebook and Craigslist, as well as through the networks of the National Association of Fibromyalgia and Related Diseases (ANFIBRO). Interested individuals accessed the survey via a link and, after providing informed consent, completed a questionnaire on the REDCap platform, which included sociodemographic information, medical history and symptom screening to confirm eligibility. Exclusion criteria included illiteracy and lack of access to a smartphone or computer to complete the questionnaire. For this analysis, 1127 individuals with chronic pain completed a REDCap‐based questionnaire, as described in *Step 4*. This sample was used to classify respondents to the definitive version of the BITEC into higher or lower treatment expectation groups. In the absence of a gold standard, we employed a Latent Class Model (LCM), which grouped the subjects into classes based on similar response patterns to the nine items of the final instrument.

The latent variable ‘Level of Expectation with Treatment’ from the TRI model is quantitative, with a scale having a mean of zero and a standard deviation of 1. To classify individuals into groups with lower or higher expectations for treatment, defining cutoff points in the latent variable became necessary. Without a ‘gold standard’ that classifies individuals into these groups, the Latent Class Model (LCA) was 6 used. The LCA model creates a categorical latent variable (each category is called a latent class) from the response profiles of individuals to the nine items of the measurement instrument, where everyone who responds to the nine items is classified into the class to which they have the highest probability of belonging (details of the LCA model are provided in the Appendix S1).

Finally, the individual will be allocated to the latent class (either lower or higher expectation for treatment) to which they have the highest probability of belonging. The higher this probability, the lower the classification uncertainty.

These LCA‐derived classes were then used as a reference standard for determining the cutoff on the IRT latent trait using the Youden Index (J = sensitivity + specificity−1), which optimises the balance between sensitivity and specificity (Youden 1950). Model selection for the nine four‐category items was guided by relative entropy and class probability distribution, with higher entropy indicating better class separation and lower uncertainty. The models were estimated using the *poLCA* (v 1.6.0.1) and *cutpoint* (v 1.1.2) packages in R. Detailed specifications of the LCA model are provided in the Appendix S1.

##### 2.1.2.4 Step 6: Assessment of Construct Validity (Discriminant Analysis)

Construct validity was examined through discriminant analysis using two complementary samples (calibration: *n* = 1127; validation sample: women with fibromyalgia, *n* = 242). This analytical strategy is theoretically grounded, as expectancy‐ and self‐regulation–based frameworks predict that symptom appraisal, perceived control and treatment beliefs vary systematically across diagnostic profiles and levels of symptom severity assessed through standardised scales. Therefore, individuals with distinct clinical patterns—and with differing severity of pain and psychosocial symptoms—are expected to be empirically distinguishable (Seligman 1975; Vlaeyen and Linton 2012; Leventhal et al. 2016).

In both samples, participants completed two validated Brazilian instruments via REDCap to capture symptom severity and cognitive‐emotional responses to pain: Brazilian Profile of Chronic Pain: Screen (Br‐PCP:S)—assesses pain intensity, functional interference and emotional burden (0–93), with higher scores reflecting greater pain impact (Caumo et al. 2013). Brazilian Pain Catastrophizing Scale (Br‐PCS)—measures helplessness, magnification and rumination (0–52), with higher scores indicating greater catastrophizing (Sehn et al. 2012).

Construct validity—defined as the extent to which an instrument measures the theoretical construct it intends to capture (Roach 2006)—was evaluated by testing whether BITEC expectation levels (low vs. high, defined by LCM cutoff) discriminated individuals according to multidimensional pain impact (Br‐PCP:S) and catastrophizing (Br‐PCS). This analysis examined whether the BITEC distinguishes between distinct clinical and cognitive‐emotional profiles, as theoretically expected.

###### 2.1.2.4.1 Calibration Sample

Used to test whether BITEC expectation levels (low vs. high, defined by LCM) discriminated among different pain diagnostic categories, as these reflect varying levels of perceived controllability and symptom complexity. Participants were categorised as nociceptive pain, fibromyalgia (primary chronic pain) and mixed/multiple chronic pain conditions other than fibromyalgia. Discriminant analysis tested whether expectation levels differed across these diagnostic groups.

###### 2.1.2.4.2 Validation Sample

For this phase, the BITEC was administered to a new sample of 242 literate women aged 18–75 years with a confirmed diagnosis of fibromyalgia according to the 2016 ACR criteria.

##### 2.1.2.5 Step 7: Development of an APP to Classify the Expectations in the Treatment Using the BITEC

An app was developed for the bedside BITEC to facilitate standardised real‐time data collection and improve decision‐making for clinicians, enhancing the reliability and validity of the scale's results. Additionally, it promotes enhanced patient engagement and participation.

### 2.2 Statistical Analysis

Descriptive statistics were calculated for all variables, and normality was assessed using the Shapiro–Wilk test. Because Br‐PCS and Br‐PCP:S scores were not normally distributed, comparisons between low‐ and high‐expectation groups were conducted using Kruskal–Wallis tests. Item calibration was performed using Samejima's Graded Response Model (R *ltm* package), retaining items based on discrimination parameters, information curves and threshold ordering; the final model consisted of nine items, with the latent trait scaled to a mean of 0 and a standard deviation of 1. Expectation levels were classified using latent class modelling, which identified two classes (low vs. high).

Construct validity was examined through two complementary approaches. First, Kruskal–Wallis tests compared catastrophizing and multidimensional pain impact across expectation levels. Second, discriminant analysis assessed whether BITEC expectation levels differentiated participants according to catastrophizing patterns and multidimensional pain domains. In the calibration sample, an additional construct‐validity test was performed using a cross‐tabulation between diagnostic categories (primary chronic pain, nociceptive pain and mixed/multiple chronic pain conditions) and BITEC expectation levels; the chi‐square test evaluated whether expectation levels differed across diagnostic groups (Campbell and Fiske 1959). Finally, an exploratory logistic regression model was applied to identify predictors of treatment expectations, controlling for collinearity among symptom severity, catastrophizing and diagnostic profile. Analyses were performed using IBM SPSS Statistics for Windows, Version 22.0 (IBM Corp., Armonk, NY, USA). All statistical tests employed a two‐tailed significance level of *p* < 0.05.

## 3 Results

### 3.1 Sample of Subjects With Chronic Pain to Select Items for Constructing the BITEC

To select the set of ‘items’ for constructing the BITEC, the sample size comprised 484 patients with chronic pain and 475 females (91.8%). The mean age and standard deviation were 48.26 (8.09), and the mean level of education was 15.16 (6.16), with a median of 14 interquartile (Q_25–75_) = 11; 19. The primary clinical diagnoses related to pain were the following: fibromyalgia 414 (85.5%); low and high back pain 40 (8.3%); osteoarthritis and rheumatoid arthritis 15 (3.1%); neuropathic pain, including trigeminal neuropathy, complex regional syndrome and neuropathy after Hansen, 11 (2.3%); and headache, 4 (0.8%).

### 3.2 Classification of Expectation Levels Using Latent Class Modelling

To classify expectation levels regarding pain treatment during scale evaluation using the LCM, the BITEC was completed by 1127 individuals with chronic pain, from a sample distinct from that used to construct the instrument. They are predominantly females, with 79.1% diagnosed with fibromyalgia and 20.9% with other chronic pain conditions. The clinical and epidemiological characteristics of the sample are presented in Table 2.

**TABLE 2:** Sample characteristics.

Characteristics	
Age (years)	48.52 (9.52)
Formal education (years)	14.37 (5.77)
Sex (female)	1307 (95.10%)
Self‐reported chronic pain diagnosis	
Fibromyalgia	891 (79.06%)
Rheumatoid arthritis, psoriatic arthritis, lupus, ankylosing spondylitis	63 (5.59%)
Neck pain	18 (1.60%)
Low back pain	46 (4.08%)
Neuropathic pain: polyneuropathy, carpal tunnel	64 (5.68%)
Osteoarthritis	41 (3.64%)
Endometriosis	4 (0.35%)
Analgesic medication use	
Codeine (yes/no)	59 (5.2%)
Methadone (yes/no)	33 (2.9%)
Tramadol	218 (19.3%)
Dipyrone (yes/no)	281 (4.80%)
Paracetamol (yes/no)	143 (12.6%)
Muscle relaxants	232 (20.5%)
Psychopharmacological medication	
Selective Serotonin Reuptake Inhibitor (yes/no)	221 (19.5%)
Duloxetine (yes/no)	416 (36.8%)
Tricyclic antidepressant (yes/no)	207 (18.3%)
Pregabalin (yes/no)	496 (43.8%)
Gabapentin (yes/no)	185 (16.3%)
Pain measures, central sensitization e pain catastrophizing	
Numerical Pain Scale (NPS 0–10)	6.53 (1.73)
Central Sensitization inventory (CSI‐BP) score	68.98 (16.18)
Pain Catastrophizing Scale – PCS	38 (10.99)

*Note:* Values are the mean (SD) or frequency (*n* = 1127).

Based on the relative entropy, Akaike Information Criterion (AIC) and Bayesian Information Criterion (BIC) values in Table 3, we chose the model with two latent classes for the low expectation category (*n* = 648) and two latent classes for the high expectation category (*n* = 479). The mean and standard deviation were −0.507 (0.560) and median (Q25–75) −0.406 [−2.64, 0.566] for the low expectation group, while for the high expectation group, they were 0.639 (0.706) and median 0.661 [Q25–75] −3.00, 1.80. BIC was used to select the best model in terms of fit and complexity, and it was more stringent in penalising complex models.

**TABLE 3:** AIC, BIC and relative entropy data according to the number of latent classes (*n* = 1127).

Number of latent classes	AIC	BIC	Relative entropy
1	22805.42	22941.16	—
2	21690.43	21966.93	0.9877037
3	21139.04	21556.31	0.9924445
4	20991.56	21549.59	0.9940797

**FIGURE 2:**
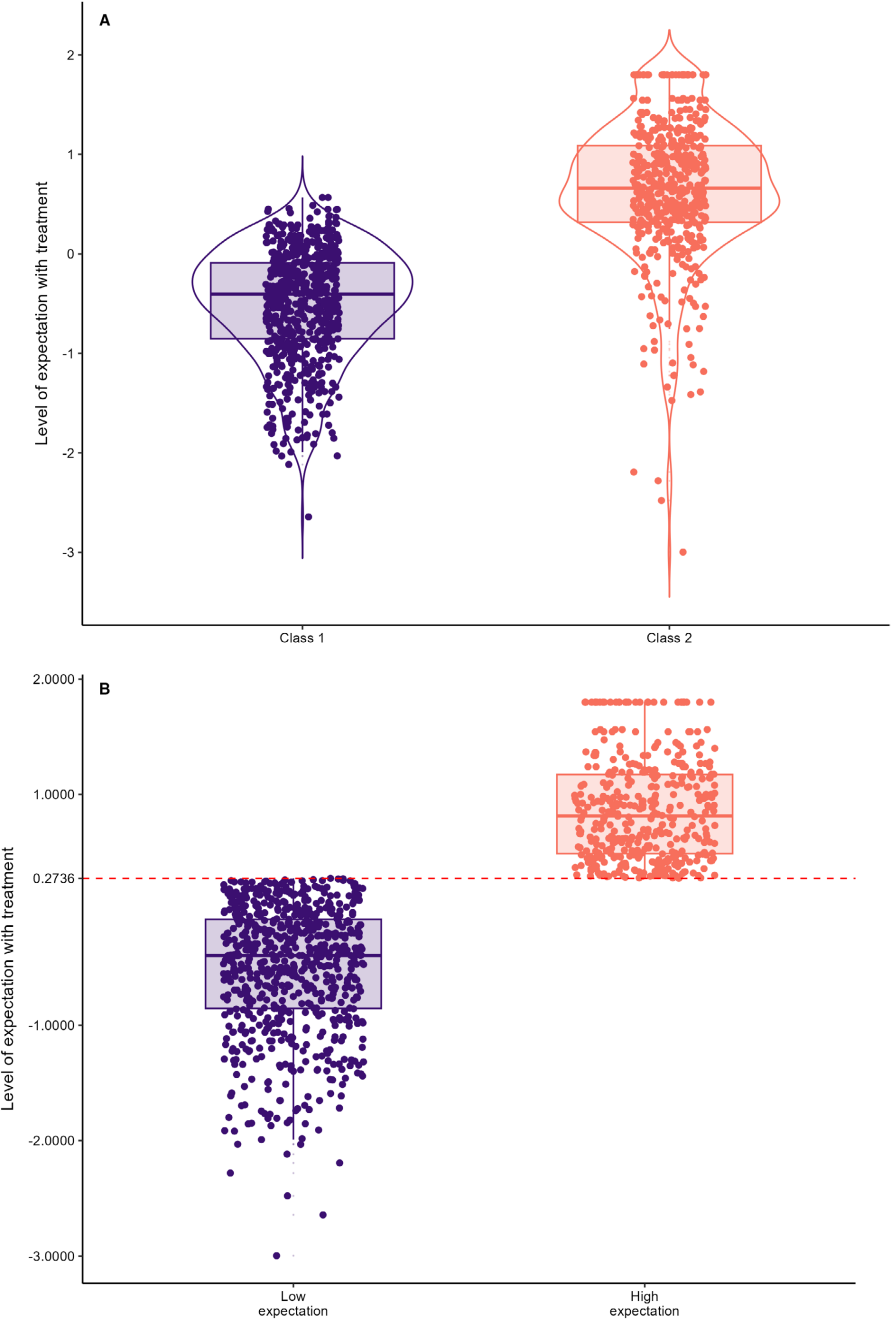
(A) Distribution of the level of expectation with the treatment (estimated by the graded response model of Samejima) according to latent class membership (determined by latent class analysis). (B) Distribution of the level of expectations with the treatment (estimated by the graded response model of Samejima) based on the latent class analysis with a cutoff point of 0.2736 and two categories: low expectations and high expectations.

The cutoff point for the TRI score was 0.2736. It was determined based on the model's classification of two latent classes as the gold standard using the Youden Index. Individuals with TRI scores up to 0.2736 were classified in the low expectation group, while those with higher TRI scores were classified in the high expectation group. TRI score data, generated from nine items with four response categories and a binary latent class, are presented in Figure 2A,B. The model showed an Area Under the Curve (AUC) = 0.915 (95% CI: 0.897–0.933), demonstrating excellent discriminative performance. At the optimal cutoff point (Youden = 0.7412), accuracy was 88.4%, with sensitivity of 78.3% (95% CI: 74.3%–81.9%) and specificity of 95.8% (95% CI: 94.0%–97.2%). Predictive values were also high, with a positive predictive value (PPV = 93.3%) and a negative predictive value (NPV = 85.7%).

**FIGURE 3:**
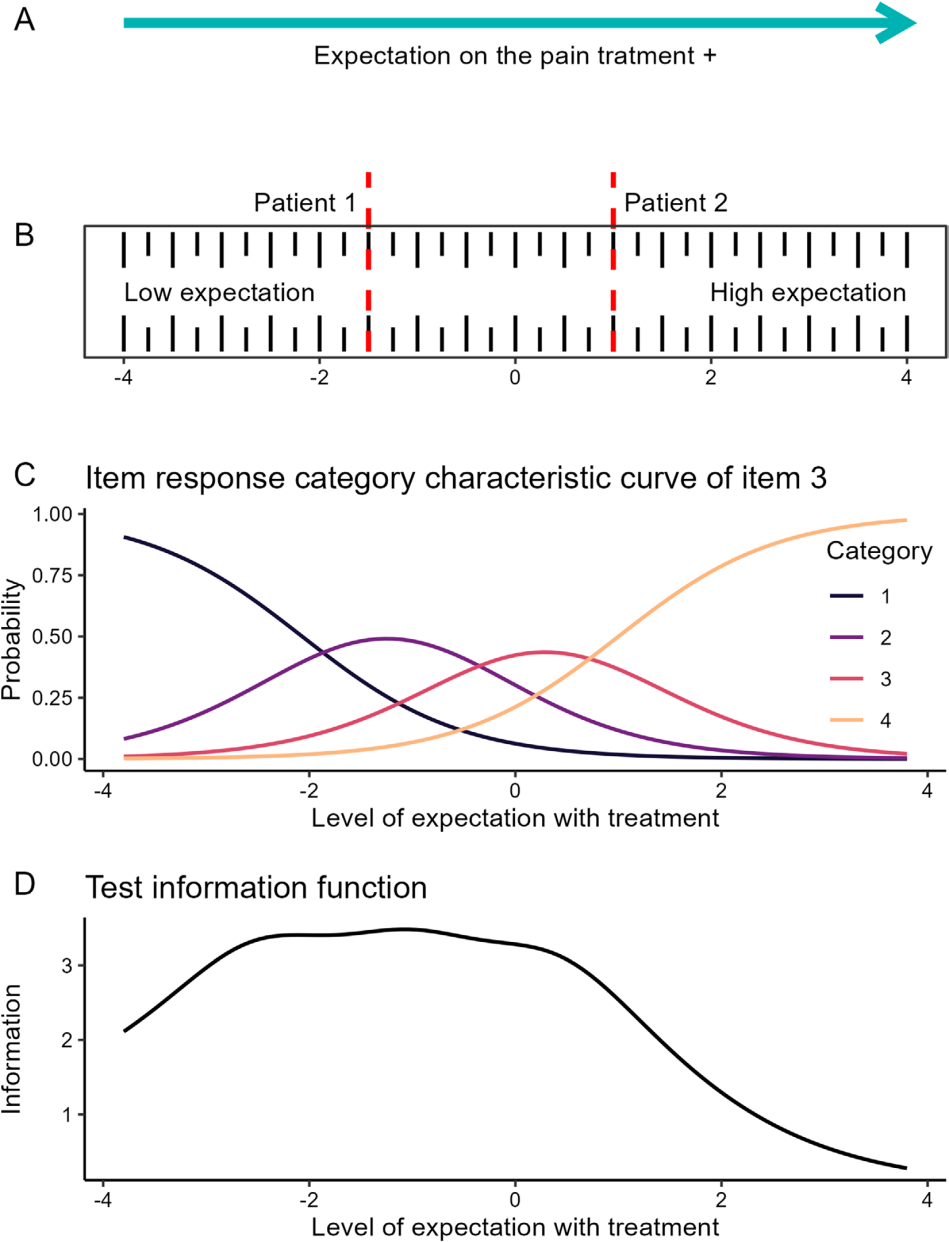
(A, B) Item response theory conceptualises the measurement scale as a ruler. Individuals with low treatment expectations are located on the left side of the scale, while those with a high level of expectation for the treatment are situated on the right side. For instance, patient A has an expected score of 1.5 standard deviations below the mean, while Patient B is 1.0 standard deviations above the mean, indicating higher expectations. (C) The lines represent the four categories on the BITEC scale: (1) Never, (2) Sometimes, (3) Almost always, and (4) Always. The x‐axis represents the level of expectation with the treatment, and the y‐axis represents the probability of responding to each response category. The further to the right on the scale, the higher the probability of endorsing a category representing a higher expectation with the treatment. (D) The test information function demonstrates the region where the proposed instrument most accurately measures the level of expectation for the treatment. In this case, the expectation will be determined with the greatest precision when it falls between the mean and the three standard deviations below.

The Item Response Theory (IRT) methodology was used to create a robust measure for the latent trait ‘Level of Expectation with the Treatment’. The results can be visualised in Figure 3A–C.

### 3.3 Assessment of Construct Validity and Discriminant Analysis

Table 4 summarises the sociodemographic and clinical characteristics of participants and highlights the discriminant profile of the BITEC both across diagnostic categories and in relation to symptom severity. In the calibration sample (*n* = 1127), 79.3% had fibromyalgia and 20.7% other pain conditions. Overall, 61.4% of participants were classified as having low and 38.6% as having high treatment expectations. High expectations were more frequent in nociceptive pain (56.1%), intermediate in fibromyalgia (42.7%), and lowest in multiple chronic pain conditions (26.9%). Compared with fibromyalgia, participants with nociceptive pain had higher odds of reporting high expectations (OR = 1.70, 95% CI 1.18–2.45, *p* = 0.004), whereas those with multiple pain diagnoses had lower odds (OR = 0.49, 95% CI 0.31–0.78, *p* = 0.002). The BITEC demonstrated discriminant validity by distinguishing expectation levels both between diagnostic profiles and by symptom burden, showing that complex pain syndromes tend to reduce perceived controllability and expectations, while greater symptom severity, regardless of diagnosis, is associated with stronger expectations for improvement.

**TABLE 4:** Sociodemographic characteristics (mean ± SD), diagnostic categories (proportions) and clinical measures (median [IQR]) according to low and high treatment expectation levels in the calibration (*n* = 1127) and validation (*n* = 242) samples.

	Calibration sample (*n* = 1127)	Validation sample (*n* = 242)
Age (years)	48.52 (SD 9.52)	50.1 (SD 8.7)
Formal education (years)	14.37 (SD 5.77)	12.1 (SD 4.8)
Sex (female)	1078 (95.10%)	242 (100%)

BITEC expectation level						
	Low (*n* = 648)	High (*n* = 479)	*p*	Low (*n* = 184)	High (*n* = 58)	*p*
Fibromyalgia (*n* = 891; 79.3%)	510 (57.2%)	381 (42.3%)				
Other pain conditions (*n* = 132; 11.6%)^a^	58 (43.9%)	74 (56.1%)				
Multiple pain conditions (*n* = 104; 9.1%)^a^, ^b^	76 (73.1%)	28 (26.9%)				

	Median and interquartile range (IQR)			Median and interquartile range (IQR)		
Brazilian Profile of Chronic Pain: Screen (Br‐PCP:S)	80 (16)	84 (12)	< 0.001	74 (17.25)	80.5 (15.5)	< 0.001
Br‐PCP:S severity	33 (7)	35 (5)	< 0.001	26 (4)	26 (4.75)	0.041
Br‐PCP:S disability	21 (7)	22 (6)	< 0.001	31 (9.25)	32 (7.5)	0.07
Br‐PCP:S emotional burden	80 (16)	84 (12)	< 0.001	18 (9)	21 (6,5)	< 0.001
Brazilian Pain Catastrophizing Scale‐total score (Br‐PCS)	39 (16)	43 (12)	< 0.001	36 (15)	43 (11.75)	< 0.001
Magnification	17 (8)	19 (6)	< 0.001	8 (4)	10 (3)	< 0.001
Helpfulness	13 (5)	14 (4)	< 0.001	16 (7.25)	18 (7)	< 0.001
Rumination	39 (16)	43 (12)	< 0.001	12 (5)	14 (4)	< 0.001

^a^ Other pain condition—presence of one chronic pain condition other than fibromyalgia (e.g., rheumatoid arthritis, psoriatic arthritis, lupus, ankylosing spondylitis, neck pain, low back pain, osteoarthritis, polyneuropathy, or carpal tunnel syndrome).

^b^ Multiple pain conditions—presence of two or more chronic pain conditions other than fibromyalgia (e.g., rheumatoid arthritis, psoriatic arthritis, lupus, ankylosing spondylitis, neck pain, low back pain, osteoarthritis, polyneuropathy, or carpal tunnel syndrome).

In the validation sample (*n* = 242, all women with fibromyalgia), 17% had comorbid neuropathic pain and 6.2% rheumatoid arthritis. Based on BITEC scores, 76% were classified as having low and 24% as having high treatment expectations.

Table 5 presents the exploratory logistic regression analysis examining the relationship between expectation levels, diagnostic profile and symptom severity. After controlling for collinearity, symptom severity and catastrophizing remained significant predictors of higher expectations, whereas diagnostic categories showed an opposite pattern, higher expectations in nociceptive pain and lower in multiple pain conditions—suggesting that expectations vary with both pain complexity and perceived functional impact.

**TABLE 5:** Predictors of treatment expectations based on symptom severity, pain diagnosis and catastrophizing (*n* = 1127).

Predictor		β (SE)	Wald	OR (95% CI)	*p*
Diagnosis category	Fibromyalgia^(reference)^	1			
	Nociceptive pain conditions	0.51 (0.17)	9.0	1.67 (1.20–2.32)	0.003
	Multiple pain conditions	−0.69 (0.21)	10.2	0.50 (0.33–0.76)	0.001
Brazilian Profile of Chronic Pain: Screen (Br‐PCP:S)		0.037 (0.009)	16.4	1.04 (1.02–1.06)	< 0.001
Brazilian Pain Catastrophizing Scale‐total score (Br‐PCS)		0.014 (0.004)	10.8	1.01 (1.00–1.02)	0.001

*Note:* β represents the regression coefficient with its standard error (SE); Wald tests indicate the significance of each predictor. Odds Ratios (OR) and their 95% confidence intervals (CI).

### 3.4 Development of an Application to Classify Credibility Levels Using BITEC

We developed an APP for classifying subjects into low and high expectation levels regarding pain treatment. The app can be accessed at this link: b‐itec.shinyapps.io/start.

## 4 Discussion

The novelty of this study lies in the development and validation of the BITEC, a brief and reliable instrument for assessing treatment expectations in chronic pain. Grounded in theoretical concepts and refined through PROM guidelines, the tool was tested in clinical samples, and IRT analysis reduced it to nine items with strong statistical performance and conceptual coherence. The BITEC demonstrated high accuracy in capturing and classifying treatment expectations, which varied consistently across clinical profiles. A diagnostic gradient was observed, with higher expectations among participants with nociceptive pain, intermediate expectations in fibromyalgia, and the lowest expectations among individuals with multiple chronic pain conditions. However, when symptom severity was examined independently of diagnosis, participants with greater multidimensional pain and psychological burden consistently reported higher expectation levels. The fibromyalgia validation sample showed a similar expectation structure, reinforcing the stability and generalizability of this classification model. By providing a robust and operational measure, the BITEC may help inform outcome prediction and support individualised treatment planning. Its concise and user‐friendly format allows bedside use and digital integration, facilitating expectation assessment while reducing potential bias in clinical decision‐making.

The evaluation of BITEC items followed recommendations established in international guidelines for the development and validation of PROMs, ensuring comprehensive assessment of treatment expectation domains. The selection of items followed COSMIN recommendations, including criteria such as content relevance, representativeness and technical quality (Terwee et al. 2018). To achieve this, a committee of experts employed the Delphi Technique, aiming for an agreement level of 80% or higher for each item. The process utilised a mixed‐methods approach, incorporating quantitative and qualitative methodologies, to obtain satisfactory content and validity outcomes. During content validity assessment, experts evaluated BITEC items according to PROMs criteria, considering comprehensibility for the target population, suitability of response options and clarity of wording (Terwee et al. 2018). The objective was to ensure that respondents easily understood items, reducing the risk of misinterpretation or confusion when applied to a sample of the target population. This approach enhanced the scale's validity and reliability, guaranteeing precise and understandable items in the BITEC index. Such clarity is crucial for accurately measuring treatment expectations and facilitating meaningful interpretation. By incorporating comprehensibility assessment using PROMs, the content validity process strengthens the scale's validity and ensures that respondents can provide accurate responses in clinical and research settings. Nevertheless, the absence of direct patient involvement in assessing item relevance from the target population's perspective represents a potential limitation regarding content validity.

According to the latent class model with two categories (low and high expectations), a cutoff point of 0.2736 achieved satisfactory accuracy, with sensitivity, specificity and an AUC of 0.91 (Hajian‐Tilaki 2013). This approach evaluates criterion validity by classifying individuals into distinct expectation levels based on their responses. Latent class modelling identifies unobserved subgroups within a population from patterns of observed responses (Lanza et al. 2013). In practice, the BITEC distinguished low and high expectations in relation to symptom severity, as measured by the Br‐PCP:S and Br‐PCS. These results indicate that the instrument captures meaningful distinctions in treatment expectations and their relationship with pain outcomes, including disability and catastrophizing, supporting its concurrent validity. To strengthen this evidence, we used an independent validation sample, reducing bias and ensuring reproducibility across populations (Fokkema and Greiff 2017).

Consistent with theoretical expectations, the BITEC demonstrated sensitivity to variation in treatment expectations across diagnostic and symptom profiles. Participants with nociceptive pain showed the highest proportion of high expectations, followed by those with fibromyalgia and multiple chronic pain conditions. This gradient supports construct validity, indicating that expectations decrease as pain becomes more complex and less predictable, consistent with models emphasising perceived control in chronic pain adaptation (Leventhal et al. 2016; Vlaeyen and Linton 2012; Benedetti 2020). Conversely, higher expectations were associated with greater pain severity and emotional distress, reflecting a motivational drive for relief described by expectancy and motivation theories. Learned helplessness further explains reduced expectations in refractory or multifactorial pain, where repeated uncontrollable symptoms erode confidence in treatment efficacy (Seligman 1975). Together, these frameworks indicate that symptom intensity and pain complexity shape the cognitive and emotional components of treatment expectancy. In this context, the BITEC captures anticipatory cognitive–affective processes rather than clinical outcomes, providing a conceptual bridge between patients' expectations and domains encompassed by the Core Outcome Sets (COS) (Patel et al. 2021). Expectations regarding pain relief and functional improvement parallel COS domains of pain intensity and interference, while coping and self‐efficacy relate to emotional well‐being and patient global assessment (Cohen et al. 2021). Accordingly, the BITEC complements clinical measures by offering a theoretically grounded perspective on how patients anticipate, engage with, and respond to treatment in complex pain conditions.

In the current study, higher pain severity and disability were associated with increased treatment expectations (Eccleston and Crombez 1999). Although expectations are multifaceted, patients with greater symptom burden and distress often exhibit heightened expectations of therapeutic benefit, consistent with evidence that suffering amplifies the perceived need for effective relief (Younger et al. 2012; Laferton et al. 2017). This pattern aligns with hypervigilant behaviour in persistent and intense chronic pain, characterised by increased attentional focus on pain sensations (Vlaeyen and Linton 2000; Crombez et al. 2005; Van Damme et al. 2004). These findings indicate an association between pain severity, psychological distress and expectations, warranting further investigation (Laferton et al. 2017). Evidence also suggests that attentional training aimed at enhancing control over pain‐related expectations may yield clinical benefits (van Ryckeghem and Crombez 2018). Nevertheless, additional research is needed to clarify the complex interplay among pain intensity, emotional distress and expectation formation (Brown and Jones 2008). Advancing this understanding may enable clinicians to better tailor therapeutic strategies and improve outcomes in chronic pain management.

Patients with high expectations also exhibited elevated pain catastrophizing across magnification, rumination and helplessness domains. The relationship between catastrophizing and expectations in chronic pain is complex and bidirectional: catastrophizing may foster negative expectations, while negative expectations may reinforce catastrophizing (Leung 2012). Excessively high expectations may also lead to disappointment when outcomes fall short, amplifying distress. Catastrophizing increases the perceived urgency for relief and is associated with exaggerated attentional engagement and impaired disengagement from pain‐related cues (Van Damme et al. 2002, 2004), reinforcing confirmation biases. These mechanisms illustrate how catastrophizing and expectations jointly influence pain perception, disability and emotional burden, with downstream effects on treatment outcomes. Healthcare providers should therefore manage expectations realistically, considering individual and contextual factors. Further research is needed to clarify underlying mechanisms and develop interventions targeting both catastrophizing and maladaptive expectations. These findings align with evidence linking catastrophizing to greater pain severity, disability and emotional distress (Quartana et al. 2009; Wertli et al. 2014), as well as lower educational attainment to greater pain impact and poorer coping responses (Gibson and Helme 2000; Blyth et al. 2001). In severe cases, high expectations may reflect a compensatory mechanism, whereby greater suffering drives stronger hope for benefit.

The BITEC is a sensitive tool for identifying treatment expectations and their influence on chronic pain outcomes; expectations can amplify placebo effects, leading to symptom relief even in the absence of physiological action (Fredrickson 2001). Neurobiological evidence supports this link, showing that positive expectations activate the endogenous opioid system and modulate pain pathways (Benedetti 2020; Wager and Atlas 2015). By capturing expectation profiles, the BITEC provides a valuable resource for understanding and leveraging placebo‐related mechanisms in pharmacological and non‐pharmacological interventions. Its relevance extends beyond measurement: the instrument may help identify psychological and contextual factors that shape treatment response, offering clinicians a means of anticipating how expectations influence therapeutic effects, including placebo modulation. Future research should validate cross‐cultural and language adaptations to broaden applicability. A limitation of the present study is the predominance of female participants, as sex differences may influence expectation patterns (Robinson et al. 2001). Although this imbalance may restrict external validity, it does not compromise the psychometric objectives, which focused on reliability and construct validity rather than representativeness (Boateng et al. 2018; Mokkink et al. 2018). Additionally, the predominance of fibromyalgia represents a potential limitation, as patients with fibromyalgia may exhibit distinct psychological profiles and pain‐related cognitive patterns compared with other chronic pain conditions, which may influence treatment expectations. In this context, the stability of the BITEC across different types of chronic pain treatments remains unknown and warrants further investigation. Despite these sample‐related limitations, recruiting participants from both clinical and community sources may introduce selection variability; this approach ensured conceptual coverage and ecological validity, capturing the diversity of expectations shaped by prior experiences and social context (Boateng et al. 2018; Mokkink et al. 2018). Notably, IRT‐based modelling enhances scale precision over Classical Test Theory by detecting differential item functioning and minimising bias from sociodemographic or cognitive factors (Embretson and Reise 2000). These features strengthen the reliability of the BITEC and its ability to inform tailored clinical strategies. Incorporating expectation assessment into practice may guide therapeutic decision‐making, improve patient communication, and optimise outcomes by accounting for the modulatory role of expectations on treatment response.

In summary, the BITEC is a brief, reliable, theory‐grounded instrument for stratifying treatment expectations in chronic pain; applicability across treatment modalities and clinical contexts warrants further investigation.

## Author Contributions

Dr Wolnei Caumo had full access to all the data in the study and takes responsibility for the integrity of the data and the accuracy of the data analysis. Concept and design: Wolnei Caumo, Bárbara Regina França. Acquisition, analysis, or interpretation of data: Wolnei Caumo, Vania Naomi Hirakata, Bárbara Regina França, Graziele Borges Bueno, Jaira Ehlers. Drafting of the manuscript: Wolnei Caumo, Rogério Boff Borges, Iraci LS da Torres, Felipe Fregni. Critical review of the manuscript for important intellectual content: Wolnei Caumo, Iraci LS da Torres, Vania Naomi Hirakata, Felipe Fregni. Statistical analysis: Wolnei Caumo, Rogério Boff Borges, Stela Maris de Jezus Castro. Obtained funding: Wolnei Caumo. Administrative, technical, or material support: Vania Naomi Hirakata, Wolnei Caumo. Supervision: Wolnei Caumo.

## Funding

This study was supported by the following Brazilian agencies: (I) Committee for the Development of Higher Education Personnel (CAPES) for material support and research grants (PROEX; grants to BRF and GBB master scholarships). (II) National Council for Scientific and Technological Development (CNPq) for research grants (I.L.S.T.: PQ no. 302345/2011‐6; WC: PQ no. 301256/2013‐6; C.B scientific initiation grant). (III) Foundation for the Support of Research at Rio Grande do Sul (FAPERGS) Ministry of Science and Technology. National Council for Scientific and Technological Development—(CNPq)/Health Secretary of state of Rio Grande do Sul, Brazil (SEARS). (IV) CHAMADA Decit/SCTIE/MS‐CNPq‐FAPERGS N° 08/2020—PROGRAMA PESQUISA PARA O SUS: gestão compartilhada em saúde—PPSUS. Postgraduate Research Group at the Hospital de Clínicas de Porto Alegre—FIPE HCPA (support project no. 2021‐0062). (V) Brazilian Innovation Agency (FINEP) (WC and ILST process no. 1245/13).

## Conflicts of Interest

The authors declare no conflicts of interest.

## Supporting information


**Appendix S1:** ejp70211‐sup‐0001‐AppendixS1.docx.

**Video S1:** ejp70211‐sup‐0002‐VideoS1.png.

## References

[ejp70211-bib-0001] Auer, C. J. , J. A. Glombiewski , B. K. Doering , et al. 2016. “Patients' Expectations Predict Surgery Outcomes: A Meta‐Analysis.” International Journal of Behavioral Medicine 23: 49–62. 10.1007/s12529-015-9500-4. 26223485

[ejp70211-bib-0002] Barth, J. , A. Kern , S. Lüthi , and C. M. Witt . 2019. “Assessment of Patients' Expectations: Development and Validation of the Expectation for Treatment Scale (ETS).” BMJ Open 9: e033578. 10.1136/bmjopen-2019-033578. PMC6585827 31213446

[ejp70211-bib-0003] Benedetti, F. 2020. Placebo Effects: Understanding the Mechanisms in Health and Disease. 3rd ed. Oxford University Press. 10.1093/oso/9780198843177.001.0001.

[ejp70211-bib-0004] Blyth, F. M. , L. M. March , A. J. Brnabic , L. R. Jorm , M. Williamson , and M. J. Cousins . 2001. “Chronic Pain in Australia: A Prevalence Study.” Pain 89: 127–134. 10.1016/S0304-3959(00)00355-9. 11166468

[ejp70211-bib-0005] Boateng, G. O. , T. B. Neilands , E. A. Frongillo , H. R. Melgar‐Quiñonez , and S. L. Young . 2018. “Best Practices for Developing and Validating Scales for Health, Social, and Behavioral Research: A Primer.” Frontiers in Public Health 6: 149. 10.3389/fpubh.2018.00149. 29942800 PMC6004510

[ejp70211-bib-0006] Bowling, A. , G. Rowe , N. Lambert , et al. 2012. “The Measurement of Patients' Expectations for Health Care.” Health Technology Assessment 16: 1–509. 10.3310/hta16300. 22747798

[ejp70211-bib-0007] Breivik, H. , B. Collett , V. Ventafridda , R. Cohen , and D. Gallacher . 2006. “Survey of Chronic Pain in Europe: Prevalence, Impact on Daily Life, and Treatment.” European Journal of Pain 10: 287–333. 10.1016/j.ejpain.2005.06.009. 16095934

[ejp70211-bib-0008] Brown, C. A. , and A. K. P. Jones . 2008. “A Role for Midcingulate Cortex in the Interruptive Effects of Pain Anticipation on Attention.” Clinical Neurophysiology 119: 2370–2379. 10.1016/j.clinph.2008.06.014. 18752995

[ejp70211-bib-0009] Campbell, D. T. , and D. W. Fiske . 1959. “Convergent and Discriminant Validation by the Multitrait–Multimethod Matrix.” Psychological Bulletin 56: 81–105. 10.1037/h0046016. 13634291

[ejp70211-bib-0010] Caumo, W. , L. S. Ruehlman , P. Karoly , et al. 2013. “Cross‐Cultural Adaptation and Validation of the Profile of Chronic Pain: Screen for a Brazilian Population.” Pain Medicine 14: 52–61. 10.1111/j.1526-4637.2012.01528.x. 23171145

[ejp70211-bib-0011] Cohen, S. P. , L. Vase , and W. M. Hooten . 2021. “Chronic Pain: An Update on Burden, Best Practices, and New Advances.” Lancet 397: 2082–2097. 10.1016/S0140-6736(21)00393-7. 34062143

[ejp70211-bib-0012] Colloca, L. , and F. G. Miller . 2011. “Harnessing the Placebo Effect: The Need for Translational Research.” Philosophical Transactions of the Royal Society, B: Biological Sciences 366: 1922–1930. 10.1098/rstb.2010.0399. PMC3130404 21576150

[ejp70211-bib-0013] Crombez, G. , S. Van Damme , and C. Eccleston . 2005. “Hypervigilance to Pain: An Experimental and Clinical Analysis.” Pain 116: 4–7. 10.1016/j.pain.2005.03.035. 15927387

[ejp70211-bib-0014] Eccleston, C. , and G. Crombez . 1999. “Pain Demands Attention: A Cognitive–Affective Model of the Interruptive Function of Pain.” Psychological Bulletin 125: 356–366. 10.1037/0033-2909.125.3.356. 10349356

[ejp70211-bib-0015] Embretson, S. E. , and S. P. Reise . 2000. Item Response Theory for Psychologists. Lawrence Erlbaum Associates. 10.1023/B:QURE.0000021503.45367.F2.

[ejp70211-bib-0016] Fitzcharles, M. A. , S. P. Cohen , D. J. Clauw , G. Littlejohn , C. Usui , and W. Häuser . 2021. “Nociplastic Pain: Towards an Understanding of Prevalent Pain Conditions.” Lancet 397: 2098–2110. 10.1016/S0140-6736(21)00392-5. 34062144

[ejp70211-bib-0017] Fokkema, M. , and S. Greiff . 2017. “How Performing PCA and CFA on the Same Data Equals Trouble: Overfitting in the Assessment of Internal Structure.” European Journal of Psychological Assessment 33: 399–402. 10.1027/1015-5759/a000460.

[ejp70211-bib-0018] Fredrickson, B. L. 2001. “The Broaden‐and‐Build Theory of Positive Emotions.” American Psychologist 56: 218–226. 10.1037/0003-066X.56.3.218. 11315248 PMC3122271

[ejp70211-bib-0019] Gibson, S. J. , and R. D. Helme . 2000. “Cognitive Factors and the Experience of Pain and Suffering in Older Persons.” Pain 85: 375–383. 10.1016/S0304-3959(99)00284-5. 10781910

[ejp70211-bib-0020] Haanstra, T. M. , T. van den Berg , R. W. Ostelo , et al. 2012. “Do Patient Expectations Influence Treatment Outcomes in Arthroplasty? A Systematic Review.” Health and Quality of Life Outcomes 10: 152. 10.1186/1477-7525-10-152. 23245187 PMC3568025

[ejp70211-bib-0021] Hajian‐Tilaki, K. 2013. “Receiver Operating Characteristic (ROC) Curve Analysis for Medical Diagnostic Test Evaluation. Caspian.” Journal of Internal Medicine 4: 627–635. 10.22088/cjim.4.2.627. PMC3755824 24009950

[ejp70211-bib-0022] Jose, A. , M. G. S. Schrooten , and J. W. S. Vlaeyen . 2017. “Treatment Expectations in Chronic Pain: The Role of Ideal and Predicted Expectations.” Pain Practice 17: 908–920. 10.1111/papr.12539.

[ejp70211-bib-0023] Kaptchuk, T. J. , and F. G. Miller . 2015. “Placebo Effects in Medicine.” New England Journal of Medicine 373: 8–9. 10.1056/NEJMp1504023. 26132938

[ejp70211-bib-0024] Kravitz, R. L. 1996. “Patients' Expectations for Medical Care: An Expanded Formulation Based on Review of the Literature.” Medical Care Research and Review 53: 3–27. 10.1177/107755879605300101. 10156434

[ejp70211-bib-0025] Laferton, J. A. C. , T. Kube , S. Salzmann , C. J. Auer , and M. C. Shedden‐Mora . 2017. “The Treatment Expectation Questionnaire (TEX‐Q): A Generic Multidimensional Scale for Assessing Patients' Treatment Expectations.” Clinical Psychology & Psychotherapy 24: 428–440. 10.1002/cpp.2002.26987691

[ejp70211-bib-0026] Laferton, J. A. C. , L. Oeltjen , K. Neubauer , D. D. Ebert , and T. Munder . 2022. “The Effects of Patients' Expectations on Surgery Outcomes in Hip and Knee Arthroplasty: A Meta‐Analysis.” Health Psychology Review 16: 50–66. 10.1080/17437199.2020.1854051. 33228474

[ejp70211-bib-0027] Lanza, S. T. , X. Tan , and B. C. Bray . 2013. “Latent Class Analysis With Distal Outcomes: A Flexible Model‐Based Approach.” Structural Equation Modeling 20: 1–26. 10.1080/10705511.2013.742377. 25419096 PMC4240499

[ejp70211-bib-0028] Leung, L. 2012. “Pain Catastrophizing: An Updated Review.” Indian Journal of Psychological Medicine 34: 204–217. 10.4103/0253-7176.106012. 23441031 PMC3573569

[ejp70211-bib-0029] Leventhal, H. , L. A. Phillips , and E. Burns . 2016. “The Common‐Sense Model of Self‐Regulation: A Dynamic Framework for Understanding Illness Self‐Management.” Journal of Behavioral Medicine 39: 935–946. 10.1007/s10865-016-9782-2. 27515801

[ejp70211-bib-0030] Mokkink, L. B. , H. C. W. de Vet , C. A. C. Prinsen , et al. 2018. “COSMIN Risk of Bias Checklist for Systematic Reviews of Patient‐Reported Outcome Measures.” Quality of Life Research 27: 1171–1179. 10.1007/s11136-017-1765-4. 29260445 PMC5891552

[ejp70211-bib-0031] Mondloch, M. V. , D. C. Cole , and J. W. Frank . 2001. “Does How You Do Depend on How You Think You'll Do? A Systematic Review of the Evidence.” Canadian Medical Association Journal 165: 174–179. 11501456 PMC81284

[ejp70211-bib-0032] Nicholas, M. , J. W. S. Vlaeyen , W. Rief , et al. 2019. “The IASP Classification of Chronic Pain for ICD‐11: Chronic Primary Pain.” Pain 160: 28–37. 10.1097/j.pain.0000000000001390. 30586068

[ejp70211-bib-0033] Page, M. G. , D. Ziemianski , M. O. Martel , and Y. Shir . 2019. “Development and Validation of the Treatment Expectations in Chronic Pain Scale.” British Journal of Health Psychology 24: 610–628. 10.1111/bjhp.12371. 30989756

[ejp70211-bib-0034] Patel, K. V. , D. Amtmann , M. P. Jensen , S. M. Smith , C. C. Veasley , and D. C. Turk . 2021. “Clinical Outcome Assessment in Clinical Trials of Chronic Pain Treatments.” Pain Reports 6: e784. 10.1097/PR9.0000000000000784. 33521482 PMC7837993

[ejp70211-bib-0035] Petrie, K. J. , and J. Weinman . 2012. “Patients' Perceptions of Their Illness: The Dynamo of Volition in Health Care.” Current Directions in Psychological Science 21: 60–65. 10.1177/0963721411429456.

[ejp70211-bib-0036] Pogatzki‐Zahn, E. M. , K. Schnabel , and U. Kaiser . 2019. “Patient‐Reported Outcome Measures for Acute and Chronic Pain: Current Knowledge and Future Directions.” Current Opinion in Anaesthesiology 32: 616–622. 10.1097/ACO.0000000000000780. 31415046

[ejp70211-bib-0037] Quartana, P. J. , C. M. Campbell , and R. R. Edwards . 2009. “Pain Catastrophizing: A Critical Review.” Expert Review of Neurotherapeutics 9: 745–758. 10.1586/ern.09.34. 19402782 PMC2696024

[ejp70211-bib-0038] Roach, K. E. 2006. “Measurement of Health Outcomes: Reliability, Validity, and Responsiveness.” Journal of Prosthetics and Orthotics 18: P8–P12. 10.1097/00008526-200601001-00003.

[ejp70211-bib-0039] Robinson, M. E. , J. L. Riley III , C. D. Myers , et al. 2001. “Gender Role Expectations of Pain: Relationship to Sex Differences in Pain.” Journal of Pain 2: 251–257. 10.1054/jpai.2001.24551. 14622803

[ejp70211-bib-0040] Samejima, F. 1969. “Estimation of Latent Ability Using a Response Pattern of Graded Scores.” Psychometrika Monograph Supplement 34: 100–114. 10.1002/j.2333-8504.1968.tb00153.x.

[ejp70211-bib-0041] Samejima, F. 1997. “Graded Response Model.” In Handbook of Modern Item Response Theory, edited by W. J. van der Linden and R. K. Hambleton , 85–100. Springer. 10.1007/978-1-4757-2691-6_5.

[ejp70211-bib-0042] Schedlowski, M. , P. Enck , W. Rief , and U. Bingel . 2015. “Neuro‐Bio‐Behavioral Mechanisms of Placebo and Nocebo Responses.” Pharmacological Reviews 67: 697–730. 10.1124/pr.114.009423. 26126649

[ejp70211-bib-0043] Sehn, F. , E. Chachamovich , L. P. Vidor , et al. 2012. “Cross‐Cultural Adaptation and Validation of the Brazilian Portuguese Version of the Pain Catastrophizing Scale.” Pain Medicine 13: 1425–1435. 10.1111/j.1526-4637.2012.01492.x. 23036076

[ejp70211-bib-0044] Seligman, M. E. P. 1975. Helplessness: On Depression, Development, and Death. W. H. Freeman.

[ejp70211-bib-0045] Terwee, C. B. , C. A. C. Prinsen , A. Chiarotto , et al. 2018. “COSMIN Methodology for Evaluating the Content Validity of Patient‐Reported Outcome Measures: A Delphi Study.” Quality of Life Research 27: 1159–1170. 10.1007/s11136-018-1829-0. 29550964 PMC5891557

[ejp70211-bib-0046] Thompson, A. G. H. , and R. Sunol . 1995. “Expectations as Determinants of Patient Satisfaction: Concepts, Theory and Evidence.” International Journal for Quality in Health Care 7: 127–141. 10.1093/intqhc/7.2.127. 7655809

[ejp70211-bib-0059] Treede, R. D. , W. Rief , A. Barke , et al. 2015. “A Classification of Chronic Pain for ICD‐11.” Pain 156, no. 6: 1003–1007. 25844555 10.1097/j.pain.0000000000000160 PMC4450869

[ejp70211-bib-0047] Van Damme, S. , G. Crombez , and C. Eccleston . 2002. “Retarded Disengagement From Pain Cues: The Effects of Pain Catastrophizing and Pain Expectancy.” Pain 100: 111–118. 10.1016/S0304-3959(02)00290-7. 12435464

[ejp70211-bib-0048] Van Damme, S. , G. Crombez , and C. Eccleston . 2004. “Disengagement From Pain: The Role of Catastrophic Thinking.” Pain 107: 70–76. 10.1016/j.pain.2003.09.023. 14715391

[ejp70211-bib-0049] van Hartingsveld, F. , R. W. Ostelo , P. Cuijpers , R. de Vos , I. I. Riphagen , and H. C. de Vet . 2010. “Treatment‐Related and Patient‐Related Expectations in Musculoskeletal Disorders: A Systematic Review.” Clinical Journal of Pain 26: 470–488. 10.1097/AJP.0b013e3181e0ffd3. 20551722

[ejp70211-bib-0050] van Ryckeghem, D. , and G. Crombez . 2018. “Pain and Attention: Toward a Motivational Account.” In Motivational Perspectives on Chronic Pain: Theory, Research, and Practice, edited by P. Karoly and G. Crombez , 211–245. Oxford University Press. 10.1093/oso/9780190627898.003.0006.

[ejp70211-bib-0051] Vlaeyen, J. W. , and S. J. Linton . 2000. “Fear‐Avoidance and Its Consequences in Chronic Musculoskeletal Pain: A State of the Art.” Pain 85: 317–332. 10.1016/S0304-3959(99)00242-0. 10781906

[ejp70211-bib-0052] Vlaeyen, J. W. S. , and S. J. Linton . 2012. “Fear‐Avoidance Model of Chronic Musculoskeletal Pain: 12 Years on.” Pain 153: 1144–1147. 10.1016/j.pain.2011.12.009. 22321917

[ejp70211-bib-0053] Wager, T. D. , and L. Y. Atlas . 2015. “The Neuroscience of Placebo Effects: Connecting Context, Learning and Health.” Nature Reviews Neuroscience 16: 403–418. 10.1038/nrn3976. 26087681 PMC6013051

[ejp70211-bib-0054] Wertli, M. M. , R. Eugster , U. Held , J. Steurer , R. Kofmehl , and S. Weiser . 2014. “Catastrophizing as a Prognostic Factor for Outcome in Patients With Low Back Pain: A Systematic Review.” Spine Journal 14: 2639–2657. 10.1016/j.spinee.2014.03.003. 24607845

[ejp70211-bib-0055] Wolfe, F. , D. J. Clauw , M. A. Fitzcharles , et al. 2016. “2016 Revisions to the 2010/2011 Fibromyalgia Diagnostic Criteria.” Seminars in Arthritis and Rheumatism 46: 319–329. 10.1016/j.semarthrit.2016.08.012. 27916278

[ejp70211-bib-0056] Youden, W. J. 1950. “Index for Rating Diagnostic Tests.” Cancer 3: 32–35. 10.1002/1097-0142(1950)3:1<32::AID-CNCR2820030106>3.0.CO;2-3. 15405679

[ejp70211-bib-0057] Younger, J. , V. Gandhi , E. Hubbard , and S. Mackey . 2012. “Development of the Stanford Expectations of Treatment Scale (SETS): A Tool for Measuring Patient Outcome Expectancy in Clinical Trials.” Clinical Trials 9: 767–776. 10.1177/1740774512465064. 23169874

[ejp70211-bib-0058] Zywiel, M. G. , A. Mahomed , R. Gandhi , A. V. Perruccio , and N. N. Mahomed . 2013. “Measuring Expectations in Orthopaedic Surgery: A Systematic Review.” Clinical Orthopaedics and Related Research 471: 3446–3456. 10.1007/s11999-013-3013-8. 23633186 PMC3792280

