# Supplementary material for: Development and Validation of the Brief Inventory of Treatment Expectations in Chronic Pain (BITEC)
Source: Eur J Pain. 2026 Jan 29;30(2):e70211. doi: 10.1002/ejp.70211 (PMC12856131; doi:10.1002/ejp.70211)
Supplement: Supplementary file 1 — Appendix S1: ejp70211‐sup‐0001‐AppendixS1.docx. [file EJP-30-0-s002.docx]

**Appendix I**

## Development and Content Validation of the Brief Inventory of Treatment Expectations in Chronic Pain (BITEC)

This study was conducted in three phases, which encompassed eight steps. ***Phase I –item* development** focused on developing the set of items for the eventual scale. This involved (1) identifying the domain(s) and generating items on a thorough literature review (1-5) and (2) assessing content validity, which entailed evaluating the content relevance, representativeness, and technical quality of each item as well as the comprehensibility based on PROM. ***Phase II – scale construction*** - involved developing the scale itself and included (3) pre-testing the questions to ensure their meaningfulness, (4) sampling and administering the survey to collect sufficient data from a sample of individuals with chronic pain, (5) reducing the number of items using Item Response Theory (IRT), and (6) classifying expectation levels using latent class modeling. ***Phase III - scale evaluation*** - focuses on evaluating the scale, including (7) assessing test validity through concurrent and discriminant validity. ***Phase IV -*** (8) Development of an app.

### Phase 1 – Item Development

#### Step 1. Generation of Items Aligned with the Concept of Treatment Expectations

The *Brief Inventory of Treatment Expectations in Chronic Pain (BITEC)* was developed through a *structured, multi-phase, theory-driven process* to ensure both conceptual coherence and content validity, consistent with international methodological frameworks for PROM development (Haynes et al., 1995; Boateng et al., 2018; COSMIN User Manual, 2018).

The conceptual basis relied on an *integrative model of treatment expectations* (Laferton et al., 2017), which encompasses both *cognitive components* (beliefs about treatment efficacy, control, and outcomes) and *behavioral components* (coping, engagement, and response to the therapeutic process). Items were designed to represent expectations of patients undergoing medical treatment for chronic pain (Jose et al., 2017) and to capture the principal theoretical domains of *outcome, structural*, and *process-related* expectations.

The BITEC was conceived as a *Patient-Reported Outcome Measure (PROM)* specifically aimed at assessing treatment expectations in clinical and research contexts (Pogatzki-Zahn et al., 2019). Its multidimensional construction integrates *internal valence*—personal beliefs, coping abilities, confidence, and perceived control—and *external valence*—expectations related to the treatment modality, the care process, and the therapeutic stimulus. This multidimensional approach enables assessment of both *how patients anticipate outcomes* and *which factors influence adherence, satisfaction, and effectiveness*.

- 1. ***Item Generation Procedures***

A clinical psychologist and a neuroscientist, both with extensive research experience in chronic pain and psychometrics, generated an initial *pool of 21 items* grounded in theoretical constructs related to treatment expectations (Hinkin, 1995). The development combined:

- *Deductive reasoning (logical partitioning):* deriving items from theoretical domains established in the integrative model of expectations.
- *Inductive reasoning (empirical classification):* refining and grouping items through content analysis, exploratory review, and expert reflection to capture subtle semantic and conceptual nuances.

This mixed deductive–inductive approach supported a broader and more representative coverage of the construct than would be possible through a purely theoretical strategy, as recommended by COSMIN Box 1a for conceptualization and domain definition.

Each candidate item underwent evaluation to confirm theoretical justification and conceptual consistency through the following steps (Haynes, 1995):

1. *Specification of purpose –* definition of the construct and intended scope of measurement.
2. *Literature review* – verification that each proposed item was supported by empirical or theoretical evidence.
3. *Preliminary conceptual definition* – articulation of the multidimensional model integrating cognitive, affective, and behavioral components.
4. *Expert judgment (face validity)* – qualitative assessment of the degree to which each item reflected the intended construct.

### *Stage 1.2 – Expert Content Validation (Delphi):*

### Initially, 21 items were developed by the authors and reviewed in two to three iterative rounds by a multidisciplinary panel of experts, to assess response processes, and ensure conceptual alignment.

To evaluate the **content validity** of the items, the Delphi method was employed. A panel of 10 expert judges reviewed three psychologists, one nurse, and one physical therapist, three physicians certified in pain management by the Brazilian Medical Board, one psychiatrist, and one neuroscientist with experience in instrument development. Among these, two psychologists and two physicians held formal certifications in pain management, ensuring methodological and clinical rigor. The expert panel reviewed each item for clarity, theoretical relevance, and representativeness of the construct. Based on their feedback, items were refined to improve conceptual precision and linguistic clarity. For the preliminary version of the BITEC scale the committee rated item using a four-point Likert scale for **content equivalence**, with the following criteria: (1) The item is not harmonically equivalent; (2) The item requires significant revision to become harmonically equivalent; (3) The item is harmonically identical, with minor changes; (4) The item is harmonically similar.

Agreement levels of ≥ 80 % among experts were deemed satisfactory for item retention, consistent with COSMIN recommendations for expert-based validation and Boateng et al. (2018). The evaluation criteria included: (i) relevance of the construct item, (ii) suitability for the target population, (iii) contextual applicability, (iv) clarity of response options, (v) appropriateness of the recall period, and (vi) comprehensibility (including linguistic clarity and cultural adaptation). After this process were selected eleven items to run the analysis

***PHASE II – SCALE DEVELOPMENT***

### *Step 3 Conceptual Classification by Valence and Pre-testing for Comprehensibility and Clarity (Adapted from COSMIN, 2018)*

3.1. Table 1 presets 11 items retained and conceptually classified according to expectation valence, based on the theoretical distinction, between internal and external expectations. *External valence,* expectations dependent on external elements or treatment context, and *internal valence*, referring to expectations grounded in personal beliefs and coping mechanisms.

# Table 1 – Theoretical Classification and Valence Type of the BITEC Items

| BITEC Item | Theoretical Category | Valence Type | IRT model status (Yes/No) |
| --- | --- | --- | --- |
| 1. I believe that this treatment will help reduce my pain. | Treatment Expectation  (Pain Reduction) | External Valence | Yes |
| 2. I hope that this treatment contributes to improving my quality of life. | Treatment Expectation  (Quality of Life) | Internal Valence | Yes |
| 3. I believe that controlling my pain depends on this treatment. | Treatment Expectation  (Control Belief) | External Valence | Yes |
| 4. My confidence in the treatment depends on how I am treated by the doctor. | Behavioral Expectation  (Doctor–Patient Interaction) | External Valence | Yes |
| 5. I believe stronger treatment leads to better pain relief. | Behavioral Expectation  (Perceived Intensity) | External Valence | Yes |
| 6. I believe I can continue the treatment even if I experience side effects. | Generalized Expectation (Tolerance) | Internal Valence | Yes |
| 7. I am committed to my treatment regardless of the difficulties I face. | Structural/Process Expectation (Commitment) | Internal Valence | Yes |
| 8. I perceive that my expectations influence the effects of the treatment. | Treatment Expectation  (Expectation Strength) | Internal Valence | Yes |
| 9. I believe this treatment has the ideal strength to relieve my symptoms. | Treatment Expectation  (Perceived Adequacy) | External Valence | Yes |
| 10. My previous treatment experiences influence the expectations I have about this treatment. | Learned Expectation (Past Experience) | External Valence | No |
| 11. My expectations about the treatment change over time. | Dynamic Expectation  (Adaptive) | Internal valence | No |

These 11 final items collectively represent a comprehensive framework of treatment-related expectations, integrating cognitive, affective, and behavioral domains within internal and external dimensions of valence. The detailed procedures described here adhere to the COSMIN Box 1a–1b standards for conceptualization and cognitive validation, ensuring transparency, reproducibility, and theoretical rigor consistent with international PROM development methodology.

## *Step 4. Item Response Theory (IRT) Calibration*

The committee of experts reevaluated each item, selecting **11 items** related to latent traits representing treatment expectations across various domains: generalized expectation ; behavior (self-efficacy and behavioral outcome expectations); treatment (treatment outcome expectations — benefits and side effects); and structural and process expectations. Items were chosen based on their content relevance to these treatment-related domains, considering attributes that could influence or explain observable behaviors or outcomes. For the IRT model, the selected 11 items evaluating treatment expectations were categorized into four groups: (0) never, (1-3) sometimes, (4-7) almost always, and (8-10) always, using a scale ranging from 0 (absence of measured content) to 10 (higher intensity). Using the Samejima Graded Response Model, nine final items were retained as they provided the greatest psychometric information about the latent trait level related to treatment expectations. However, items 10 and 11 were excluded from the IRT model because they did not contribute additional discriminative information.

**Assumption of unidimensionality in unidimensional IRT models:**

The Samejima Graded Response Model requires two assumptions for appropriate use: local independence and unidimensionality—that is, a single latent trait must account for item responses. (Samejima, 1969; Samejima, 1997). These assumptions are related, as local independence is satisfied when the instrument is unidimensional (Andrade et al., 2000; Hays et al., 2000). The unidimensionality assumption can be relaxed to sufficient unidimensionality, meaning that the instrument may include minor secondary dimensions as long as a predominant factor exists, typically defined as the first factor explaining at least 20% of the total variance (Bernstein et al., 2007; Chan et al., 2004; McHorney & Cohen, 2000). The assessment of sufficient unidimensionality for the proposed measurement instrument was conducted using exploratory factor analysis based on the polychoric correlation matrix, given the ordinal polytomous nature of the items, implemented with the *psych* package (version 2.5.3) in R.

The first latent dimension accounted for 34.4% of the total variance across the nine items, indicating that the assumption of sufficient unidimensionality was met. The estimate parameter are presented in **table 2**.

## Table 2 – Item Parameter Estimates under the Graded Response Model for BITEC Items (Training Sample)

|  | Discrimination (SE) | b1 | b2 | b3 |
| --- | --- | --- | --- | --- |
| 1. I believe that this treatment will help reduce my pain. | 0.803 (0.078) | -3.680 (0.343) | -0.506 (0.146) | 1.281 (0.215) |
| 2. I hope that this treatment contributes to improving my quality of life. | 1.116 (0.112) | -4.434 (0.436) | -2.551 (0.368) | -1.059 (0.278) |
| 3. I believe that controlling my pain depends on this treatment. | 1.310 (0.099) | -2.069 (0.139) | -0.427 (0.093) | 1.000 (0.143) |
| 4. My confidence in the treatment depends on how I am treated by the doctor. | 1.237 (0.101) | -3.093 (0.228) | -1.502 (0.195) | 0.178 (0.125) |
| 5. I believe stronger treatment leads to better pain relief. | 1.923 (0.150) | -2.495 (0.148) | -1.024 (0.161) | 0.392 (0.107) |
| 6. I believe I can continue the treatment even if I experience side effects. | 1.059 (0.088) | -2.795 (0.215) | -0.873 (0.139) | 0.933 (0.137) |
| 7. I am committed to my treatment regardless of the difficulties I face. | 0.953 (0.098) | -4.891 (0.504) | -2.716 (0.396) | -0.501 (0.269) |
| 8. I perceive that my expectations influence the effects of the treatment. | 0.994 (0.087) | -3.712 (0.313) | -1.399 (0.209) | 0.636 (0.156) |
| 9. I believe this treatment has the ideal strength to relieve my symptoms. | 0.650 (0.078) | -6.819 (0.860) | -3.122 (0.537) | 0.480 (0.363) |

*bᵢ,ₖ = difficulty parameter for the k-th category of item i
SE = standard error*

The nine items retained in the IRT model capture the essential dimensions of treatment expectations within a biopsychosocial framework. Items 1, 3, 5, and 9 reflect the **biological domain**, addressing beliefs about treatment efficacy, pain control, and perceived adequacy. Items 2, 6, 7, and 8 represent the **psychological domain**, encompassing hope, self-efficacy, commitment, and metacognitive awareness. Item 4 covers the **social/interactional domain**, referring to the therapeutic relationship. Collectively, these items integrate cognitive, affective, and behavioral aspects of treatment expectations, providing a balanced representation of the biological and psychological components, with partial but meaningful coverage of the social dimension.

*Graded Response Model of Samejima (1969)*

The probability that an individual chooses a category $k$, or greater than $k$, of item $i$ is:

$$P_{i,k}^{+}\left( \theta_{j} \right)=\frac{1}{1+e^{-Da_{i}\left( \theta_{j}-b_{i,k} \right)}}$$

$i=1,2,\ldots,9 j=1,2,\ldots,n k=1, 2, 3$

where, $\theta_{j}\to level of expectation with the treatment of the individual j$

$a_{i}\to SLOPE parameter of item i$ $b_{i,k}\to difficulty parameter of the k-th category of item i$

Being $P_{i,1}^{+}\left( \theta_{j} \right)=1$ , $P_{i,3+1}^{+}\left( \theta_{j} \right)=0$ e $b_{i;1}\leq b_{i;2}\leq b_{i;3}$ .

Therefore, the probability of an individual choosing exactly category $k$ of item $i$ is:

$$P_{i,k}\left( \theta_{j} \right)=\frac{1}{1+e^{-Da_{i}\left( \theta_{j}-b_{i,k} \right)}}-\frac{1}{1+e^{-Da_{i}\left( \theta_{j}-b_{i,k+1} \right)}}$$

2. The LCA model is defined as according to reference [(25)](https://www.zotero.org/google-docs/?aessmJ):

$$P\left( Y=y \right)=\sum_{c=1}^{2} \gamma_{c}\prod_{m=1}^{9} \prod_{k=1}^{4} \rho_{mk|c}^{I\left( y_{m}=k \right)}$$

Let Y denote the possible response profiles to the nine items of the measurement instrument YYY; P(Y=y) is the probability of an individual presenting a specific response profile y; I(y_m=k) is the indicator function, which equals one if the individual responded to category k of item m; γ_c is the population proportion (or prevalence) of individuals in category c of the latent variable 'Class of Expectation with Treatment," with c = 1 (individuals with lower expectation for treatment) or c = 2 (individuals with higher expectation for treatment); _(mk|c) is the probability of responding to category k for the m-th item conditioned on class c, i.e., according to the category of expectation with treatment in which the individual is found, it indicates the probability of responding to category k of the m-th item. After fitting the latent class model, it is possible to estimate the posterior probability (p_ic), i.e., the probability of individual i belonging to class c according to their response profile to the nine items of the measurement instrument.

YYY:$p_{ic}=\frac{\left( \prod_{m=1}^{9} \prod_{k=1}^{4} \rho_{mk|c}^{I\left( y_{m}=k \right)} \right)\gamma_{c}}{\sum_{c=1}^{2} \gamma_{c}\prod_{m=1}^{9} \prod_{k=1}^{4} \rho_{mk|c}^{I\left( y_{m}=k \right)}}$

Finally, the individual will be allocated to the latent class (either lower or higher expectation for treatment) to which they have the highest probability of belonging. The higher this probability, the lower the classification uncertainty.

The relative entropy criteria were used to select the best latent class model (LCM) for items with four categories. Relative entropy is calculated by considering the probability distribution of the latent classes, aiming to quantify the balance between these classes. Higher relative entropy indicates less uncertainty about the latent classes, indicating less difficulty distinguishing between the model's different classes. The Akaike Information Criterion (AIC) and Bayesian Information Criterion (BIC) were used to help choose the best-fitted model.

The nine items measuring the latent trait of treatment expectation, generated by the Samejima Graded Response Model, form a continuous quantitative variable with a mean of zero and a standard deviation of 1. The interpretation of this measure is based on the number of standard deviations above or below the mean of zero. The Samejima Graded Response Model was fitted using R's ltm package, version 1.2-0

***Step 6: Latent Class Model (LCM) to classify the expectation levels***

For the latent class modeling (LCM) analyses, we selected adult participants with chronic pain using an online survey. Participants were recruited through public announcements and online advertisements (e.g., Facebook, Craigslist). The survey was administered via REDCap® and disseminated to the target audience through multiple channels, including a patient network from the Tertiary Chronic Pain Treatment Clinic at the Hospital de Clínicas de Porto Alegre (HCPA). Recruitment efforts included internal and external communications via email, the hospital website, social media platforms, and outreach by the Pain and Neuromodulation Laboratory. A total of 1127 participants completed all study procedures.

To classify respondents according to their level of treatment expectations based on responses to the definitive version of the BITEC scale, we applied a Latent Class Model (LCM). This approach was selected due to the absence of a gold standard for categorizing individuals as having low or high treatment expectations. The LCM groups participants into classes based on similar response patterns across the nine items of the final instrument.

In parallel, the latent trait variable "Level of Expectation with Treatment" was obtained from the Item Response Theory (IRT) model, which is a continuous measure with a mean of 0 and a standard deviation of 1. To classify individuals into groups with lower or higher expectations, it was necessary to define cut-off points on this latent continuum. Because there was no external gold standard for such classification, the Latent Class Analysis (LCA) was used to generate a categorical latent variable, wherein each participant was assigned to a latent class (i.e., lower or higher expectation) based on their highest posterior probability of class membership. Everyone was ultimately allocated to the class to which they had the highest posterior probability. The greater this probability, the lower the classification uncertainty. The resulting latent classes from the LCA were considered a reference standard for determining the cutoff point on the continuous latent trait scale (IRT-based).

To define this cutoff point, the Youden Index was applied, which is defined as *J = sensitivity + specificity – 1*. This method maximizes the balance between sensitivity and specificity in differentiating between the LCA-derived classes. The poLCA package (version 1.6.0.1) in R was used to estimate the latent class model, and the cutpoint package (version 1.1.2) in R was used to compute the Youden Index.

To select the optimal LCM for the 9 items (each with four response categories), model selection was based on relative entropy and the distribution of class probabilities. Higher relative entropy values indicate better class separation, meaning less classification uncertainty and greater clarity in distinguishing between latent classes.

References

1. Andrade DF de, Tavares HR, Valle R da C. Teoria de Resposta ao Item: Conceitos e Aplicações [Internet]. São Paulo: SINAPE; 2000. Available from: <https://docs.ufpr.br/~aanjos/CE095/LivroTRI_DALTON.pdf>
2. Hays RD, Morales LS, Reise SP. Item Response Theory and health outcomes measurement in the 21st Century. Med Care. 2000;38(9 Suppl. II):28–42.
3. Bernstein IH, Rush AJ, Carmody TJ, Woo A, Trivedi MH. Clinical vs. self-report versions of the quick inventory of depressive symptomatology in a public sector sample. J Psychiatr Res. 2007;41(3–4):239–46.
4. Chan KS, Orlando M, Ghosh-Dastidar B, Duan N, Sherbourne CD. The interview mode effect on the Center for Epidemiological Studies Depression (CES-D) Scale An item response theory analysis. Med Care. 2004;42(3):281–9.
5. Mchorney CA, Cohen AS. Equating health status measures with item response theory Illustrations with functional status items. Med Care. 2000;38(9 Suppl II):43–59.
6. Samejima, F. (1969). Estimation of Latent Ability Using a Response Pattern of Graded Scores (Psychometric Monograph No. 17). Richmond, VA: Psychometric Society.
7. Samejima, F. (1997). Graded Response Model. In: van der Linden, W.J., Hambleton, R.K. (eds) Handbook of Modern Item Response Theory. Springer, New York, NY. <https://doi.org/10.1007/978-1-4757-2691-6_5>
