# Supplementary figures and images for: Development and Validation of the Brief Inventory of Treatment Expectations in Chronic Pain (BITEC)
Source: Eur J Pain. 2026 Jan 29;30(2):e70211. doi: 10.1002/ejp.70211 (PMC12856131; doi:10.1002/ejp.70211)

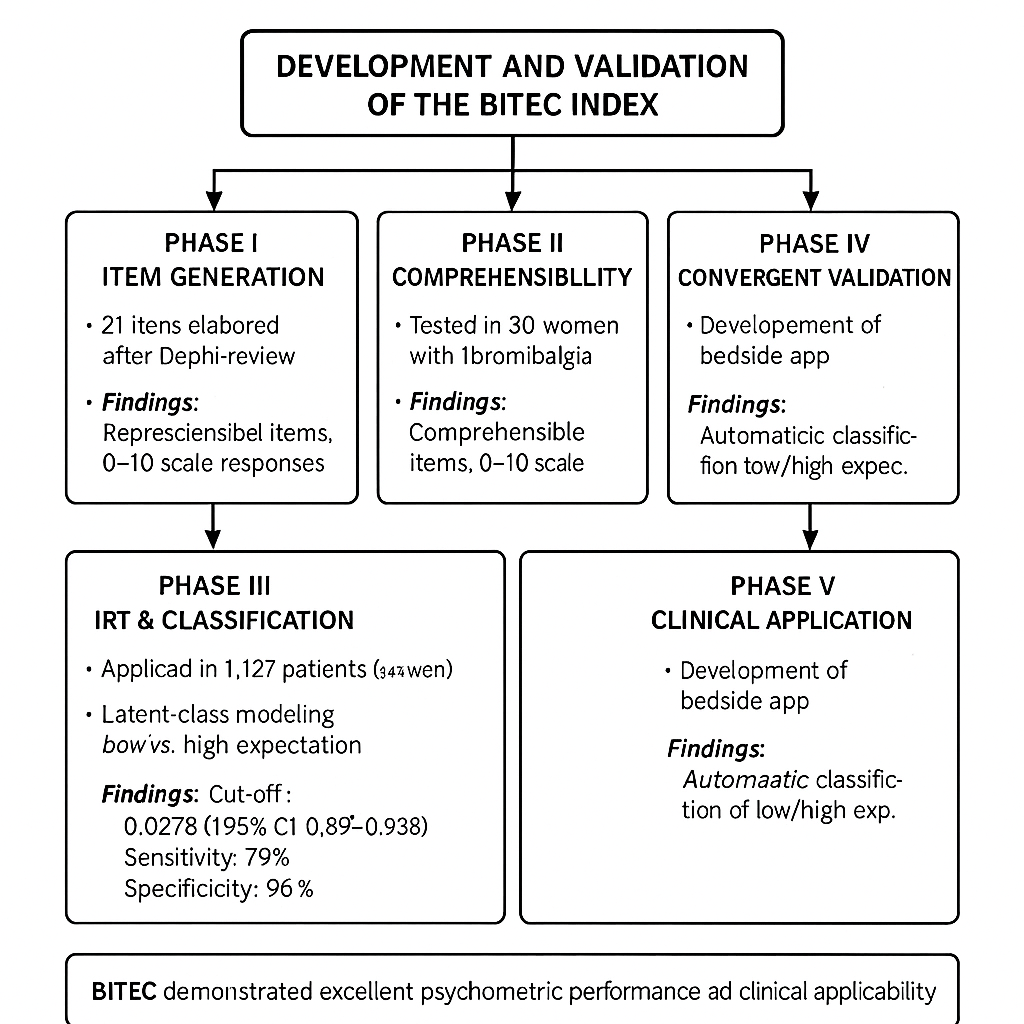

Supplement: Supplementary file 2 — Video S1: ejp70211‐sup‐0002‐VideoS1.png. [file EJP-30-0-s001.png]
